# Planetary Health and Health Education in Brazil: Towards Better Trained Future Health Professionals

**DOI:** 10.3390/ijerph191610041

**Published:** 2022-08-15

**Authors:** Walter Leal Filho, João Henrique Paulino Pires Eustachio, Alberto Paucar-Caceres, Melissa Franchini Cavalcanti-Bandos, Cintia Nunes, Carlos Vílchez-Román, Silvia Quispe-Prieto, Luciana Londero Brandli

**Affiliations:** 1 European School of Sustainability Science and Research (ESSSR), Hamburg University of Applied Sciences, 21033 Hamburg, Germany; 2 Department of Natural Sciences, Manchester Metropolitan University, Chester Street, Manchester M15 6BH, UK; 3 School of Economics, Business Administration and Accounting at Ribeirão Preto, University of São Paulo (USP), Avenida dos Bandeirantes 3900, Ribeirão Preto 14040-900, SP, Brazil; 4 All Saints Campus, Manchester Metropolitan University, Manchester M15 6BH, UK; 5 Centro Universitario Municipal de Franca, Franca 14401-426, SP, Brazil; 6 CENTRUM Católica Graduate Business School (CCGBS), Pontificia Universidad Católica del Perú (PUCP), Lima 15023, Peru; 7 Escuela Profesional de Enfermería, Facultad de Ciencias de la Salud, Universidad Nacional Jorge Basadre Grohmann, Tacna 23000, Peru; 8 Graduate Program in Civil and Environmental Engineering, University of Passo Fundo, Campus I-BR 285, Passo Fundo 99052-900, RS, Brazil

**Keywords:** planetary health, higher education, health education, bibliometric analysis, Brazil

## Abstract

Brazil is Latin America’s largest country and has a strong economy, but it is also characterised by many inequalities. These are very conspicuous in the health sector, particularly in health education, which is expected to modernise according to the planetary health (PH) perspective. This paper describes the health education scenario in Brazil and undertakes an analysis of the postgraduate health programmes and policies in place, identifying the extent to which these support the cause of PH. To achieve this goal, this paper deploys a bibliometric analysis to gain a better understanding of the research streams related to higher education and PH. In addition, it presents and discusses selected case studies in the field and cross-checks documents from the Brazilian Ministry of Education against five domains of PH in education. The results indicate that despite some progress to date and the fact that some programmes are in place, there is a perceived need for policies and efforts from education organisations towards connecting PH principles in the education of current and future health professionals.

## 1. The Health Sector and Planetary Health in Brazil

Planetary health (PH) is an emerging concept as humans continue to damage the earth systems with severe consequences to human beings and their health. The concept arose in 2013 when Horton identified that the idea of “global health” should be extended towards the planet and not solely on health issues and solutions [1].

PH emerges as a concept that aims to investigate Anthropocene problems in environmental and social systems and how they are connected to the health of the systems. In this sense, PH is related to “the achievement of the highest attainable standard of health, well-being, and equity worldwide through judicious attention to the human systems—political, economic, and social—that shape the future of humanity and the earth’s natural systems that define the safe environmental limits within which humanity can flourish” [2]. To put it simply, PH is the health of human civilisation and the state of the natural systems on which it depends.

PH is important in all regions of the world, but especially in Brazil, it is worthy of attention. Of the total of approximately 213.3 million people living in Brazil in 2021, the Brazilian Statistics Institute estimates that 24.1% live below the threshold of poverty of USD 5.5 a day [3]. In addition, when it comes to other dimensions of PH and sustainable development, Brazil presents several challenges, such as sanitation, plastic emissions and a high rate of deforestation, that would considerably impact the health of the population and ecosystems [4,5,6]. Moreover, since the sustainability problems are shared among all countries globally, Brazil needs attention due to its continental characteristics and the potential to negatively impact several earth systems. In other words, guaranteeing PH on the Brazilian territory could promote the sustainability of natural systems and people’s health within Brazilian borders and, as a consequence, create positive externalities worldwide.

The shared responsibility among countries is not the only challenge in pursuing PH. The literature also argues about the importance of several industries in contributing towards PH and sustainable development [7], whereby the health sector became expected to contribute to PH more systemically because of its evident connections among public health, holistic medicine and some medical conditions that emerged or increased due to sustainability problems, such as pollution, climate change, sanitation and poverty [8,9].

Despite the vibrant discussion on the connection between PH issues and the health sector, little research has been conducted to understand in a comprehensive way how health education programmes are prepared to tackle PH challenges. Moreover, the authors found no studies that aimed to assess how health programmes are adherent to PH principles. In this sense, this study builds on previous work that reported on the importance of higher education health programmes in contributing to PH [10,11] and fosters the discussion and practice in two ways: (1) shedding light on the existing research streams of higher education and PH through a bibliometric assessment and (2) assessing the status of postgraduate health education programmes in Brazil by analysing the documents from the Brazilian Ministry of Education against the five domains of the PH education framework developed by Guzmán and colleagues—the Anthropocene and health, movement building and systems change, systems thinking and complexity, equity and social justice and interconnection within nature [10].

## 2. Health Education in Brazil

**Figure 1:**
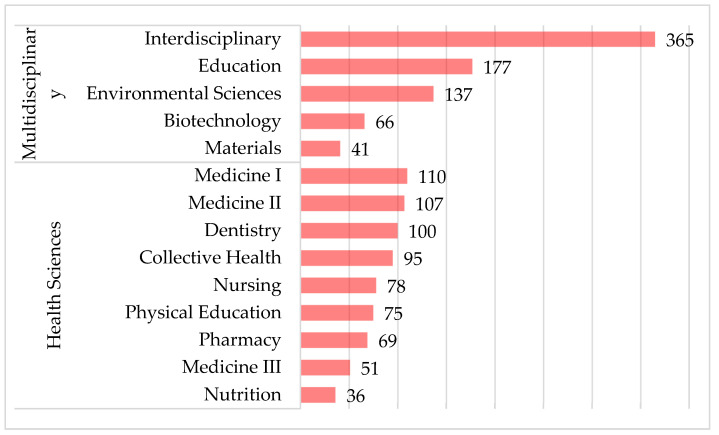
Health sciences and multidisciplinary programmes. Source: CAPES [12].

The Federal Ministry of Education coordinates health education in Brazil through a bureau in charge of accrediting and evaluating postgraduate programmes: the Coordination for the Improvement of Higher Education Personnel [12]. In terms of postgraduate education, Brazil has 4607 programmes recognised by CAPES that are divided into nine areas: agrarian sciences, applied social sciences, biological sciences, engineering, exact and earth sciences, health sciences, human sciences, linguistics and arts and multidisciplinary [13]. Of the total, 721 are embedded in the health sciences, and 786 belong to the multidisciplinary category. Figure 1 shows the number of programmes in each area.

The health sciences category, in turn, entails nine areas. Medicine appears at the top of the list with 268 programmes, considering the sum of medicine I, II and III, differentiated by the medical specialities on which these programmes focus. In sequence, dentistry appears next with 100 postgraduate programmes, followed by collective health (95), nursing (78), physical education (75), pharmacy (69) and nutrition (36) [13].

The multidisciplinary category is a separate one that contains five areas: interdisciplinary, education, environmental sciences, biotechnology and materials. The area with the most postgraduate programmes is the interdisciplinary field (365 programmes), which exists due to the unfortunate problems that emerge in the contemporary world and that require the interconnection of several areas of knowledge. In sequence, education appears with 177, environmental sciences 137, biotechnology 66 and materials 41 programmes.

This landscape suggests some connections with the PH perspective for both graduate and postgraduate programmes. However, it is still unclear how deeply these programmes are fully equipped to deal with PH challenges and, therefore, address the gap raised by Shaw and colleagues that “health professions graduates are not prepared” to deal with the urgent challenges of the UN´s SDGs and the principles of PH [14].

## 3. Materials and Methods

**Figure 2:**
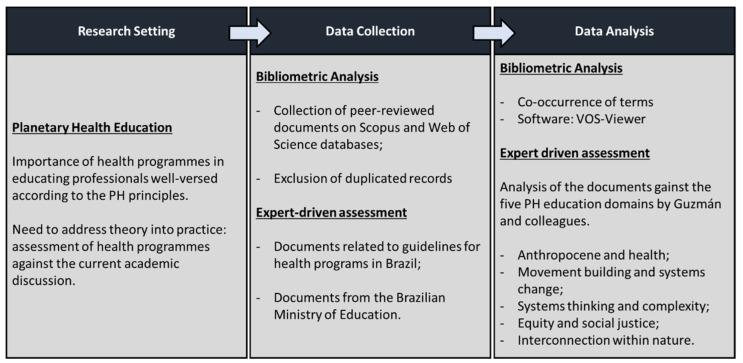
Research setting [10].

This research relies on bibliometric analysis to understand the connections between PH and higher education and an expert-driven assessment of the postgraduate programmes in Brazil in order to understand the extent to which these programmes are connected to PH. Figure 2 summarises the research background and the deployed data collection and analysis strategies.

For the bibliometric analysis, a search string was created to find related peer-reviewed documents in both the Web of Science [15] and Scopus [16] databases, which are considered to have the broadest coverage of documents in social, physical, health and life sciences. Table 1 describes the terms used and the number of records obtained from each of the scientific databases used.

The string was created to search relevant documents in Scopus and Web of Science (respecting the particularities of each database) using terms related to PH, education and possible variations commonly found in the literature, such as university, higher education institutions (HEIs) or higher education. The search was performed in January 2022 and returned 110 documents on the Web of Science and 136 on the Scopus database. The results from both databases were analysed in order to exclude duplicated records (e.g., documents that appear simultaneously in both databases) before proceeding with the bibliometric analysis.

Regarding the inclusion criteria, the authors included all document types (e.g., article, letter, review, etc.) to comprehensively overview the PH publications available in the scientific mainstream. Concerning exclusion criteria, we filtered out the publications without abstracts because that textual data was used to carry out the bibliometric analysis. After this selection, 211 records (Scopus = 113, WoS = 98) were obtained and the results were saved in two comma-separated value (CSV) files. After merging the CSV files, the authors eliminated duplicated records (*n* = 71), which accounted for 34% of the filtered records. As a result, the final dataset had 140 records. Given that PH is an emerging field, the authors found it was a reasonable dataset size for the study.

For the data analysis stage, a bibliometric analysis was adopted to understand the commonly discussed topics in the literature related to higher education and PH. Text mining was conducted using VOSviewer to identify the co-occurrence of terms [17], and the results are presented by a network graph, in which the diameters of bubbles refer to the frequency of terms, and the width of connections between bubbles corresponds to the strength of the connection between two terms. In addition, terms with a high co-occurrence frequency are likely to appear close to each other, corresponding to a thematic cluster [18]. We used a stopword file to filter out terms (e.g., article, author, paper, part, study, etc.) that did not contribute to understanding thematic clusters obtained with VOSviewer. The software’s exploring techniques (cluster analysis and multidimensional scaling) let the authors identify the thematic clusters. For measuring the connection strength of the most relevant terms with each other, we obtained two centrality measures: in-betweenness (measures the link strength of each node) and closeness (measures the level of connection for each term).

Finally, the authors also relied on an expert-driven assessment of the status of health education in Brazil by exploring the documents related to the postgraduate programmes of the Brazilian Ministry of Education against the five domains of the PH education framework: interconnection with nature, systems thinking and complexity, equity and social justice, the Anthropocene and health, movement building and systems change and systems thinking and complexity [10]. The results and discussion are presented in the next section.

## 4. Results and Discussion

### 4.1. Bibliometric Assessment

**Figure 3:**
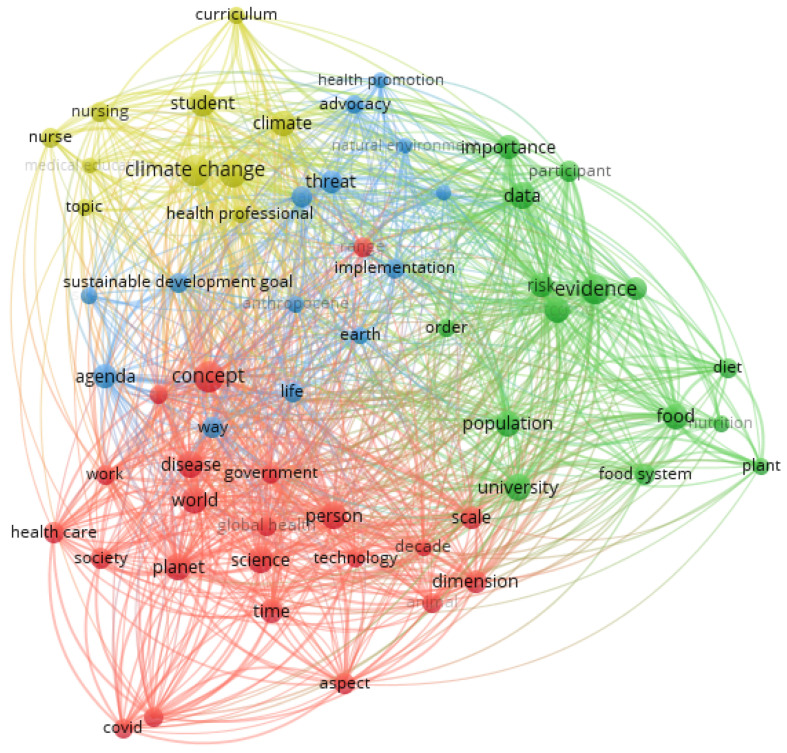
Co-occurrence analysis of PH and terms related to HEIs. Note: Co-occurrence of the terms—VOSviewer output.

Figure 3 shows the findings of the co-occurrence analysis regarding the search string developed using the terms PH and health education. Overall, the results returned four main clusters, which can be considered research streams in which experts have conducted the discussion in this field. In addition, the results indicate that the field is systemic, going in the same direction as the previous definition of PH, which is related to the “interconnections between the health of person and place at all scales” [9].

The red cluster is the biggest in the number of terms. It evidences the connection between PH, education and the healthcare systems. In a broad sense, the studies in this cluster try to unveil the main aspects and domains of PH and how these could be incorporated into healthcare systems and health education. For example, some authors argue about a framework for sustainable health systems based on a couple of principles, such as reducing demand for health services, matching the supply of health services to demanding and reducing emissions from the supply of health services and optimising the efficiency and environmental performance of care delivery [8]. Other studies go further and discuss the main dimensions of PH that should be considered for health education or even develop a framework that could serve as a guide for implementing PH in the curricula of health programmes based on five primary spheres: interconnection with nature, the Anthropocene and health, movement building and systems change, systems thinking and complexity and equity and social justice [10]. In this sense, much attention is given to how it could be possible to embed PH into clinical education since understanding the interactions between human health environments has become essential [9,19,20] and how the training of health professionals (e.g., medicine and nursing) should be adapted to consider PH aspects [20].

The green cluster, in turn, focuses the central discussion on planet health and food systems, evidencing the need to provide changes to the food systems and policies in place so as to preserve the environment and protect human health. In this sense, research in this cluster aims to raise attention to the need for overconsumption policies, the reduction of animal-based foods and an increase in plant-based foods [21]. In addition, this cluster relates to another major problem also connected to PH, which is the overweight and obese condition, evidencing that society should be aware of sustainable and healthy diets as well as the apparent demand for food policies that could contribute to public health and reduce environmental impacts [22]. Moreover, other authors also discuss the importance of food security governance and its positive effects on equitable and sustainable food systems, such as reducing the threat of infectious diseases, reducing the environmental footprint and enhancing nutrition [23,24].

The blue cluster, in turn, contains terms related to PH and sustainable development goals (SDGs, threat, implementation, Earth and life). Research in this field is broad and tries to explore the connections in how pursuing the SDGs could benefit PH and vice versa [25]. Examples of studies belonging to this cluster evidence the synergies among SDG 3 (good health and well-being), SDG 12 (sustainable consumption and production) and SDG 15 (life on land) to PH [21]; how financial systems could contribute to PH [26]; urbanisation as a threat to biodiversity, natural systems and PH [27]; and many other studies that try to understand directly and indirectly the connections of the sustainable development challenges to PH [28].

Finally, the yellow cluster is comprised of relevant terms explicitly connected to climate change, students, curriculum, nursing and health professionals. In this perspective, the discussion embedded in this cluster is twofold. The first refers to the importance of health professionals’ awareness of sustainable development challenges, especially climate change and the connection between social and natural systems, underlining that PH is a concept that should not be ignored or postponed by health professionals [9,29,30]. The second discussion is related to the extent to which health programmes should innovate their programmes by identifying ways of educating health professionals with a set of competencies and knowledge according to the PH principles, making them able to contribute to a resilient climate future [10,11,19,20,31].

### 4.2. PH and Health Education in Brazil

PH education should be implemented across all levels of education and training. However, given its novelty, it is more likely that this emerging field first appears in HEIs, more specifically, postgraduate programmes, which usually engage in new research fields before they are mainstreamed. This section provides an overview of postgraduate programmes in Brazil, focusing on interdisciplinary studies related to the foundational domains of the PH education framework [10].

If an HEI proposes the creation of a new programme to the Coordination for the Improvement of Higher Education Personnel (CAPES) and the programme does not fall within the indicated knowledge area, the request is denied. This may explain why it is difficult to find health sciences programmes that focus on the environment. In fact, when analysing the latest evaluation reports [13], only one programme of 112 in the environmental sciences [32] area was on the environment, health and sustainability provided by the University of São Paulo. For the collective health area, only two programmes focused on environment and health: public health and environment (FIOCRUZ) and the programme health, environment and work (Federal University of Bahia).

In addition, the inclusion of new “areas of knowledge” makes it possible to open new innovative research fields for HEIs. This is the case in the interdisciplinary studies area, which since its creation in 1999, the number of courses/programmes submitted for appreciation has increased [33].

Of the 368 programmes in interdisciplinary studies that exist today in Brazil and are evaluated by CAPES [33], a number of them are connected to the foundational domains of the PH education framework [10]. Given the research foci of these interdisciplinary programmes, it would be a timely endeavour for them to include the PH education framework in their curricula specifically.

In the Brazilian HEI system, a programme in the field of PH would fall within the interdisciplinary studies knowledge area. Interdisciplinary studies programmes focus on solving urgent problems that require aggregation of knowledge [33]. CAPES sees the mission of interdisciplinary studies not as an attempt to overthrow the existing division of disciplines but as a tool to encourage postgraduate training to approach problems that cannot be solved from the point of view of a sole discipline.

The Document of Interdisciplinary Area [33] guides the courses in evaluating aspects every 4 years. Despite not mentioning the PH approach, there is a concern for attending to the challenges of this century through interdisciplinary courses: “the Interdisciplinary Area aims to allow program proposals to find space and offer new strategies to address contemporary challenges” [33].

Table 2 shows an analysis of interdisciplinary courses in Brazil from the perspective of five domains of the PH education framework [10]. The courses have been clustered according to their main concepts and study areas. The assumption used by the authors is that these courses, when considering their aims and approaches, are contributing in different ways to PH education.

### 4.3. Considerations about the Future of the Interdisciplinary Area Associated with PH

The last CAPES report about the interdisciplinary studies area shows that there is space for experimenting with new approximations between different areas of knowledge, enabling the constitution of approaches to original theoretical–methodological studies and the development of innovative research, teaching and extension practices [33]. This approach connects directly with the sense of urgency presented in the framework report on PH [10]. Therefore, there is a necessary connection between postgraduate programmes and PH issues because the interconnectivity between human societies and the biosphere generates a need for scientific solutions and collective action.

In addition, important recommendations were made related to PH such as: enhancing the monitoring and tackling of tropical diseases, strengthening primary health, basic sanitisation and waste management, moving towards a national energy production system with a low level of coal utilisation, stopping illegal deforestation and reducing biomass burning, enhancing air quality through the adoption of an air quality index and enhancing environmental legislation, improving food and nutritional security and implementing a sustainable development unit in the Brazilian health system [34]. These issues are relevant to moving society towards better PH, and they become key aspects to be incorporated into health and interdisciplinary education and research, generating practical solutions for Brazil in the short term.

Moreover, following the CAPES report, it appears that one of the most significant challenges of this century is the (re)connection of knowledge, a perspective that expands in the interdisciplinary area, as it is a space for innovation in the organisation of postgraduate education and research and a space that induces the interdisciplinary and humanist formation of students, professors and researchers [33]. This training focuses on developing and adopting an interdisciplinary attitude in their different teaching, research and extension practices, including the necessary insertion of the scientific and technological production generated. We associated this necessary training of students, professors and researchers with the five dimensions of PH in postgraduate programmes: interconnection within nature, movement building and system change, the Anthropocene and health, equity and justice and system thinking and complexity [10].

## 5. Conclusions

The bibliometric analysis identified four main clusters in which authors engaged in PH and higher education discussion. The first cluster is related to how the concept and aspects of PH could be incorporated into the healthcare system and education; the second connects PH and food systems and food security with food policy that could benefit human health and protect the environment at the same time; the third cluster presents a broad discussion about sustainable development and highlights how the UN´s SDGs could benefit PH and vice versa. The last cluster explores how PH should be implemented in the health education curricula, the competencies related to PH and how to make health professionals aware of the PH issues in their work.

The status of integration of PH in health education in Brazil is currently in its initial stages. For this reason, most postgraduate programmes still do not fully capitalise on the potential of handling PH issues. This is not uncommon in developing countries since engagement in new research fields is slow, and it takes time until they are mainstreamed. In addition, this shows why the interdisciplinary studies area, which is also related to health education, is characterised by accelerated growth in connecting to PH paradigms. Therefore, it is suggested that both undergraduate and postgraduate health education programmes should pay greater attention to the need to promote PH principles.

The implications of this study are threefold. Firstly, it illustrates the potential contribution that health programmes that address PH dimensions may provide to society by educating future health professionals and also connecting sustainability and PH principles. Secondly, it provides theoretical evidence about the importance of adopting PH to develop competencies and knowledge when adopting a multidisciplinary and transdisciplinarity perspective in health education programmes. Finally, this study draws attention to the importance of implementing PH not only in Brazil but also in the health education systems of other Latin American countries, as they share some similarities in terms of challenges related to public health and health education (e.g., sanitation, food security, tropical diseases). This also includes aspects related to climate change and other concerns related to achieving the UN Agenda 2030 of sustainable development in general.

However, to yield the expected benefits and ensure that PH is more present in university-based health education programmes in Brazil, there is an urgent need for policies at HEIs that recognise the usefulness of and need for embedding PH principles in health programmes in Brazil. Apart from support from policymakers, this requires:

- the design of curricular guidelines that adhere to the PH domains;
- that managerial staff at universities are aware of the interconnectedness of PH and the UN´s SDGs so as to maximise its impact;
- that programme coordinators work towards including PH in graduate/postgraduate programmes.

Finally, it is also important that teaching staff make efforts to connect their disciplines to PH issues, showing their students that climate change, global-scale pollution of air, water and soil and the degradation of forests, rivers, coastal and marine systems are also matters of concern to the health professions.

## Figures and Tables

**Table 1:** Search string and number of documents.

Database	Search String	Number of Documents
Web of Science	TS = ((“Planetary Health”) AND (“academi*” OR “universit*” OR “higher education institution*” OR “HEI*” OR “higher education” OR “education”))	110
Scopus	TITLE-ABS-KEY((“Planetary Health”) AND (“academi*” OR “universit*” OR “higher education institut*” OR “HEI*” OR “higher education” OR “education”))	136

**Table 2:** Assessment of PH domains in health education in Brazil.

The Planetary Education Framework [10]	Evidence in the Brazilian Health Education (Names of the Courses/Programmes in Interdisciplinary Areas)		Key Concepts and Areas of Study
Interconnection Within Nature	Cultural Diversity and Social Inclusion Bioethics (2 programmes) Human Rights and Public Policies	Human Rights Interdisciplinary Studies on Women, Gender and Feminism Social Policies and Citizenship	These courses have in common the aim to work with one or more of these study areas or concepts: traditional knowledge systems; nature connectedness (human–nature connectedness); ecological identity; epistemological diversity and humility; worldviews, from Animism to Cartesianism; two-eyed seeing; kincentric; resilience; and Pachamama, Gaia, and other similar concepts.
The Anthropocene and Health	Health and Environment (5 programmes) Environmental Health Environmental Sciences and Health Biodiversity, Environment and Health Health, Society and Environment Work, Health and Environment Sustainable Development and Quality of Life	Society, Environment and Quality of Life Health and Human Development Health and Human Aging (11 programmes) Health Surveillance (2 programmes) Sciences Applied to Health Biosciences and Health Health and Work	These courses have in common the aim to work with one or more of these study areas or concepts: the social and environmental determinants of health, the Anthropocene and related concepts, Anthropocentric and ecocentric, globalisation, demographic transition, epidemiological transition, planetary boundaries, and ecological footprint.
Systems Thinking and Complexity	Professional Health Education (2 programmes) Health Education (2 programmes) Health Information and Communication Dissemination of Science, Technology and Health Scientific and Cultural Dissemination	Decision and Health Models Human Ecology and Socio-Environmental Management Interdisciplinary Studies in the University Sustainable Management Systems Sustainable Development and Extensão	These courses have in common a focus on one or more of these study areas or concepts: epistemological diversity and humility; transdisciplinarity; uncertainty; implicit/explicit bias and self-awareness; unintended/unexpected consequences; and scale, including geographical scale (local/regional/global—micro/meso/macro) and temporal scale (past/present/future—top priority/low priority—urgent/elective).
Equity and Social Justice	Preservation and Management of The Cultural Heritage of Sciences and Health Preservation of Cultural Heritage Society and Culture in the Amazon Health, Society, and Endemics in the Amazon Ethnic and African Studies Culture and Territorialities	Ethnic and Racial Relations Ethnic Relations and Contemporaneity Cultural Processes and Manifestations Management and Health in The Amazon Natural Resources Management and Local Development in the Amazon Science, Innovation and Techno	These courses have in common a focus on one or more of these study areas or concepts: accessibility; equity and inequity; social, distributive, intergenerational and multispecies justice; the rights of nature; cultural humility; empathy; privilege; and racism and oppression.
Movement Building and Systems Change	Community Development Social Management, Education and Regional Development Development and Public Policies Agroecology and Sustainable Rural Development Sustainable Environmental Systems Sustainability Environment and Sustainability Natural Resources Energy and Environment	Environment and Rural Development Territorial Development and Environment Environmental Science and Technology Environmental Sciences (2 programmes) Environmental Technologies and Innovations Sociobiodiversity and Sustainable Technologies Rural Development and Business Management. Agri-Food Sustainable Development Practices	These courses have in common the aim to work with one or more of these study areas or concepts: urgency and hope; strategic visioning; theory of change; the spectrum of allies; advocacy; entrepreneurship; innovation; empowerment, autonomy and agency; collaboration—participation; inclusivity/diversity; capacity building; and resilience.
